# Psychosocial impact of implantable cardioverter defibrillators (ICD) in young adults with Tetralogy of Fallot

**DOI:** 10.1007/s00392-012-0420-x

**Published:** 2012-02-08

**Authors:** Petra Opić, Elisabeth M. W. J. Utens, Philip Moons, Dominic A. M. J. Theuns, Arie P. J. van Dijk, Elke S. Hoendermis, Hubert W. Vliegen, Natasja M. S. de Groot, Maarten Witsenburg, Martin Schalij, Jolien W. Roos-Hesselink

**Affiliations:** 1 Department of Cardiology, Thoraxcenter, Erasmus Medical Center, Rotterdam, The Netherlands; 2 Department of Child and Adolescent Psychiatry and Psychology, Erasmus Medical Center- Sophia Children’s Hospital, Room KP2865, P.O. Box 2060 – KP2, 3000 CB Rotterdam, The Netherlands; 3 Center for Health Services and Nursing Research, Katholieke Universiteit Leuven, Leuven, Belgium; 4 Department of Cardiology, University Medical Center Nijmegen, Nijmegen, The Netherlands; 5 Department of Cardiology, University Medical Center Groningen, University of Groningen, Groningen, The Netherlands; 6 Department of Cardiology, Leiden University Medical Center, Leiden, The Netherlands

**Keywords:** Congenital heart disease, ICD, Quality of life, Tetralogy of Fallot, Inappropriate shocks

## Abstract

**Objective:**

To investigate the psychosocial impact of having an implantable cardioverter defibrillator (ICD) in adults with Tetralogy of Fallot (ToF).

**Methods:**

Included were 26 ToF-patients with an ICD (age 44 ± 12 years), and two control groups consisting of 28 ToF-patients without an ICD (age 40 ± 10 years) and a group of 35 ICD-patients of older age without ToF (age 72.0 ± 8 years). This last control group was chosen to represent the “older general ICD population” with acquired heart disease seen at the out-patient clinic. Psychosocial functioning encompassed daily functioning, subjective health status, quality of life, anxiety, depression, coping and social support.

**Results:**

ToF-patients with ICD showed diminished psychosocial functioning in comparison to ToF-patients without ICD. This was reflected by diminished subjectively perceived physical functioning (*p* = 0.01), general health perception (*p* < 0.01) and a lower satisfaction with life (*p* = 0.02). In comparison to older ICD-patients, ToF-patients with ICD showed less satisfaction with life (*p* = 0.03), experienced more anxiety (*p* = 0.01) and showed less favourable coping styles, although physical functioning was better for ToF-patients with ICD than for older ICD-patients (*p* = 0.01). More inappropriate shocks were found in ToF-patients with ICD compared to the older ICD-patients.

**Conclusion:**

In patients with ToF, ICD implantation had a major impact on psychosocial functioning which should be taken into account when considering ICD implantation in these young patients. To help improve psychosocial functioning, psychological counselling attuned to the specific needs of these patients may be useful.

## Introduction

The leading cause of mortality in adult patients with congenital heart disease (ConHD) is sudden cardiac death (SCD) [1]. In comparison with the general population, an adult patient with ConHD has a 25–100 fold increased risk to die as a result of SCD [2]. Implantable cardioverter defibrillators (ICDs) are being used as therapy for patients that are at high risk for developing, or have survived a life-threatening cardiac arrhythmia [3]. However, the indication of ICD therapy in patients with ConHD is still a matter of debate. A recent publication from our group investigating the efficacy of ICD therapy in ConHD patients demonstrated that 23% of all patients received an appropriate shock, and 41% of the investigated patients received at least one inappropriate shock [4]. This inappropriate shock rate is higher than in other patient groups. Other studies reported an inappropriate shock rate of around 25%, and an appropriate shock rate of around 22–30% in ConHD patients [3].

Congenital heart disease patients not only have to cope with an increased risk to die as a result of SCD [2]. In addition, the implantation of an ICD with associated inappropriate shocks, which occur when the patient is fully conscious, may cause anxiety for shock, anxiety for premature death and stress, hereby worsening the psychological problems [6, 7]. Patients receiving ICD therapy may show reduced quality of life, subjective health status and diminished social functioning. Anxiety and fear for ICD discharge does not only affect the patient, it can also influence the behaviour of relatives and friends surrounding the patient. Sometimes these “significant others” experience fear and anxiety, which may have a cumulative anxiety arousing effect on the patient [8]. The aim of this study was to investigate the psychosocial impact that ICD therapy has in (young) adults with Tetralogy of Fallot (ToF). We chose patients with ToF because most of the ICD implantations in the ConHD population occur in these patients [3].

Two control groups were selected; the first control group consisted of ToF-patients without an ICD. We selected these patients to investigate the impact of the ICD while holding the groups comparable on cardiac diagnosis and hemodynamic burden. The second control group consisted of older acquired heart disease ICD patients. This group represents the “general” ICD population as seen in an outpatient clinic. By choosing this group, we could make a comparison with a “general” average ICD patient. Age may be a factor in acceptance of an ICD. Also, knowledge about the psychosocial functioning of patients with an ICD comes from studies focusing on these “regular ICD-patients”. These patients are older and have acquired heart disease. The psychosocial problems seen in these older patients may be quite different than those seen in younger ConHD patients receiving ICD therapy. Young patients do not only experience the problems associated with the ICD but also carry the burden of having grown-up with a congenital heart defect. They also experience more inappropriate shocks than non-ConHD patients [4]. These shocks may lead to anxiety, psychosocial problems and avoidance behaviour, limiting patients in social contacts, and leisure time activities. In addition, overprotective parents of ConHD patients may be a limiting factor as well. Therefore, we hypothesized that the psychosocial impact of receiving ICD therapy in young ConHD patients may be more substantial.

Finally, we wanted to compare whether styles of coping and adjustment to ICD therapy differed between younger ConHD patients with an ICD (ToF + ICD) and older patients with acquired heart disease receiving ICD therapy (ICD), when adjusted for the time-period of receiving ICD therapy.

## Methods

### Study design and population

This is a cross-sectional, multicentre study.

#### Inclusion criteria

Our database consisted of three groups. The first group consisted of Fallot-patients with ICD (ToF + ICD). This population was selected using the CONCOR [9] registry in The Netherlands and a Belgian tertiary care centre adult ConHD database. The CONCOR registry is a nationwide database consisting of adult patients with ConHD, including medical history. The selection of this patient sample is described in detail in the paper of Yap et al. [4]. This group was used as the study population. The second group consisted of Fallot-patients without ICD (ToF) and was also identified using the CONCOR registry. This ToF group consisted of 28 patients without significant differences in age, sex and NYHA class compared to the study group ToF + ICD. We used this group as our first control group. The third group (ICD) consisted of 35 older ICD-patients with another form of heart disease, mainly ischaemic heart disease. These patients did not have ConHD. This group was identified using the Erasmus MC ICD registry, and did not show significant differences regarding gender compared to the ToF + ICD group. We used this last group as our second control group. For all selected patients, data were collected form medical records, with permission of the patients and physicians.

This study was approved by the institutional ethical committees. All patients provided informed consent before participating in this study.

#### Patient sample

Of the 44 eligible patients from the ToF + ICD group, 13 were lost to follow-up and 3 patients died before inclusion in this study. The present patient sample consisted of the remaining 28 adults of whom two refused to participate, resulting in a response rate of 93% for the ToF + ICD group. The mean age of this group was 44 years (±12 years).

There were no differences between participants and non-participants on age, age at Fallot correction, shunt before correction, reoperations, age at ICD implantation, follow-up time, indication for ICD implantation, NYHA class or the amount of shocks.

In order to ensure that ICDs of patients are programed optimally, patients visited the outpatient clinic every 6 months, or sooner if they had complaints. The functionality of the ICD device was assessed by skilled technicians and adapted if necessary. All appropriate and inappropriate shocks were recorded.

#### Indications for ICD implantation in ToF-patients

The index event before ICD implantation was spontaneous sustained ventricular tachycardia (VT) in 14 patients (54%), cardiac arrest in 5 patients (19%), (pre) syncope in 5 patients (19%) and other in 2 patients (8%).

#### Assessment procedure

All patients were approached uniformly and signed an informed consent before participating. All patients completed the questionnaires at home and returned them by mail. Missing items were retrieved by means of a telephone call.

### Instruments

The psychological examination consisted of the following questionnaires.

#### Biographical characteristics

A semi-structured questionnaire was designed to assess biographical variables such as nationality, living conditions, marital-, educational- and occupational status [10]. The educational attainments were evaluated excluding two patients living in institutions because of psychosocial problems.

#### Subjective health status

The subjective health status was assessed by the SF-36 Health Survey [11]. Good reliability and validity for the Dutch version of the SF-36 has been reported [12].

#### Satisfaction with Life Scale

The Satisfaction with Life Scale (SWLS) was used as an indicator of the satisfaction with life. This scale has been proven psychometrically sound to be used in ConHD patients [13].

#### Linear Analogue Scale Quality of Life

The Linear Analogue Scale (LAS) was used to measure self-perceived quality of life. The LAS has been proven valid, reliable and responsive for the ConHD population [13].

#### Hospital Anxiety and Depression Scale (HADS)

This scale measures the presence and severity of anxiety and depression in patients. The HADS has been validated for the general Dutch population and is stable across medical settings and age groups [14, 15].

#### Utrecht Coping List

The Utrecht Coping List (UCL) is a reliable and standardised self-report questionnaire of coping styles. The satisfactory validity of the UCL has been described elsewhere [16]. Construct validity and predictive validity has been examined for the UCL.

#### Implanted Devices Adjustment Scale

The Implanted Device Adjustment Scale (IDAS) measures the psychological adjustment of a patient to an implanted (ICD) pacemaker. The IDAS has been described valid, reliable and responsive [17].

#### Perceived Social Support Scale

The Perceived Social Support Scale 12 item version (PSSS12) measures the interactions and discrepancies that people experience in receiving social support from their direct environment [18].

### Statistical methods

Biographical characteristics were analysed using Chi-Square tests. Because of the skewed nature of the data, Mann–Whitney *U* tests were used to test for differences between the ToF + ICD group versus both control groups on all questionnaires. Comparison with normative data was made using Students’ *t* tests, since raw data for the norm groups were not available, and only mean and standard deviations were available. Descriptive statistics of continuous variables are expressed as medians with quartiles. Stepwise multiple regression was used to correct for age at implantation, and the higher age in the ICD group. The data were analysed using the statistical package SPSS PSAW 17.0.2 ENG for Windows, Release 17.0.2 (Mar 11, 2009).

## Results

### Population characteristics

#### Biographical characteristics

The main biographical and medical characteristics for the three groups are outlined in Table 1. No differences in gender were found between the three groups. Patients from the ToF + ICD group were living significantly less often on their own compared to patients from the ICD group. Patients in the ToF + ICD group more often had no relationship and were less often widowed than the older ICD patients. After adjusting for higher age at implantation in the ICD group, it appeared that this variable did not have an effect on the SF-36 results.

**Table 1:** Biographical and medical characteristics

	ToF + ICD (*N* = 26)	ToF (*N* = 28)	ICD (*N* = 35)	Group effect	
				ToF + ICD vs. ToF (*p* value)	ToF + ICD vs. ICD (*p* value)
Response rate	93%		88%		
Gender					
Male	15 (57.7%)	19 (67.9%)	28 (80.0%)	0.4	0.06
Age^a^	44 (±11.58)	40 (±10.26)	72 (±8.28)	0.1	**<0.0001**
Nationality					
Dutch	18 (69.2%)	28 (100.0%)	35 (100.0%)	**<0.01**	**<0.0001**
Belgian	8 (30.8%)	0 (0.0%)	0 (0.0%)	**<0.01**	**0.001**
Living conditions					
With parents	2 (7.7%)	3 (10.7%)	0 (0.0%)	1.0	0.1
Living alone	21 (80.8%)	22 (78.6%)	34 (97.1%)	0.1	**0.02**
Institution/home replacement	3 (11.5%)	3 (10.7%)	1 (2.9%)	1.0	0.1
Marital status					
No relationship	8 (30.8%)	8 (28.6%)	2 (5.7%)	0.9	**0.02**
Stable relationship	0 (0.0%)	2 (7.1%)	0 (0.0%)	0.5	–
Cohabitant	1 (3.8%)	6 (21.4%)	0 (0.0%)	0.1	0.2
Married	15 (57.7%)	9 (32.1%)	21 (60.0%)	0.06	0.9
Divorced	1 (3.8%)	0 (0.0%)	1 (2.9%)	0.5	0.8
Cohabitant or married after divorce	1 (3.8%)	1 (3.6%)	1 (2.9%)	1.0	0.8
Widowed	0 (0.0%)	2 (7.1%)	8 (22.9%)	0.5	**0.02**
Stable relationship or married after being widowed	0 (0.0%)	0 (0.0%)	2 (5.7%)	–	0.2
Occupational level					
Elementary	0 (0.0%)	1 (3.6%)	0 (0.0%)	1.0	–
Lower	3 (11.5%)	6 (21.4%)	0 (0.0%)	1.0	0.5
Average	7 (26.9%)	8 (28.6%)	6 (17.1%)	0.3	0.3
Higher	3 (11.5%)	8 (28.6%)	1 (2.9%)	0.5	1.0
No job/missing data	13 (50.0%)	5 (17.9%)	28 (80.0%)		
Educational attainment^b^					
Lower	2 (7.7%)	2 (7.1%)	8 (22.9%)	1.0	0.1
Average	17 (65.4%)	21 (75.0%)	20 (57.1%)	0.7	0.3
Higher	5 (19.2%)	5 (17.9%)	5 (14.3%)	1.0	0.5
Other	0 (0.0%)	0 (0.0%)	1 (2.9%)		
Missing	1 (3.8%)	0 (0.0%)	0 (0.0%)		
Medical data					
Age fallot correction (years)^a^	9.9 (±10.80)	5.9 (±3.91)	–	0.1	–
Shunt before correction	8 (30.8%)	13 (48.1%)	–	0.2	–
Reoperations	15 (57.7%)	12 (44.4%)	–	0.3	–
Age at implantation (years)^a^	36.5 (±11.25)	–	64.7 (±8.19)	–	**<0.0001**
Follow-up (years)^a^	7.9 (±3.73)	–	7.4 (±2.08)	–	0.5
Indication for ICD					
Primary prevention	6 (23.1%)	–	13 (37.1%)	–	0.2
Secondary prevention	20 (76.9%)	–	22 (62.9%)	–	0.2
NYHA-class					
I	19 (73.1%)	24 (85.7%)	9 (25.7%)	0.2	**<0.0001**
II	5 (19.2%)	3 (10.7%)	24 (68.6%)	0.5	**<0.0001**
III	2 (7.7%)	1 (3.6%)	2 (5.7%)	0.6	1.0
Brady pacing^c^	7 (33.3%)	(0.0%)	15 (42.9%)		0.5
QRS duration^a^	176 (±27.2)	150 (±25.2)	138 (±39.3)	**<0.01**	**<0.0001**
RV dilatation					
None	2 (10.0%)	6 (21.4%)		0.195	
Moderate	11 (55.0%)	18 (64.3%)			
Severe	7 (35.0%)	4 (14.3%)			
RV function					
Good	10 (50.0%)	26 (92.9%)		**<0.01**	
Reduced	10 (50.0%)	2 (7.1%)			
Pulmonary regurgitation					
None/mild	12 (63.2%)	13 (46.4%)		0.05	
Moderate	6 (31.6%)	5 (17.9%)			
Severe	1 (5.3%)	10 (35.7%)			
Inappropriate ICD shocks	10 (38.5%)		5 (14.3%)		**0.03**
Appropriate ICD shocks	2 (7.7%)		11 (31.4%)		**0.03**

ToF + ICD, patients with Tetralogy of Fallot with an ICD

ToF, patients with Tetralogy of Fallot (without an ICD)

ICD, patients with ICD (without congenital heart disease)

*NYHA* New York Heart Association class

^a^Data are presented as mean (±SD)

^b^Excluding two patients living in institutions because of psychosocial problems

^c^There were 5 patients in the ToF + ICD group of which the ECG could not be examined

The bold numbers in the text indicate significant results

No significant differences were found between the three groups with respect to occupational level or educational attainment. In all three groups, the majority had an average educational attainment.

#### Medical characteristics

No significant difference was found regarding age, the amount of surgical procedures or re-operations between the ToF + ICD and the ToF group. As we selected older ICD patients to represent the “normal” ICD population as our second control group, the patients in the ToF + ICD group were younger than those in the ICD group. Also, patients in the ToF + ICD group were (as planned) significantly older at the time of surgical Fallot correction than patients from the ToF group. Both indication for ICD implantation (primary and secondary) and follow-up time after ICD did not differ between the two groups. The majority of patients in the ICD group were NYHA class II resulting in a significant difference with regard to the ToF + ICD group and the ToF group (majority NYHA class I). When comparing QRS duration, a significant difference was found between all three groups. Group ToF + ICD had the highest QRS duration (176 ms) followed by the ToF group (150 ms) and the ICD group (138 ms). No difference was found between the ToF + ICD and ToF groups when comparing right ventricular dilatation. Right ventricular function was significantly worse in the ToF + ICD group compared to the ToF group (*p* < 0.01). Remarkably, patients from the ToF + ICD group less often had severe pulmonary regurgitation compared to patients form the ToF group. When analyzing the occurrence of appropriate and inappropriate shocks, a significantly higher incidence of inappropriate shocks was observed in the ToF + ICD group versus the ICD group (*p* = 0.03). Also, the absolute number of inappropriate shocks was higher in the ToF + ICD group (*p* = 0.03) and also the amount of appropriate shocks was higher in the ToF + ICD group (*p* = 0.03).

### Scores on instruments (see Table 2)

#### Subjective health status (SF36)

On all SF-36 scales except for one, the median for the ToF + ICD group was lower, indicating more unfavourable outcomes, than for the ToF group. Two significant group effects between the ToF + ICD group versus the ToF group were found: patients from the ToF + ICD group scored significantly lower on physical functioning compared to the ToF group (*p* = 0.01) and also on general health perceptions patients from the ToF + ICD group scored significantly lower than the ToF group (*p* < 0.01). When comparing the ToF + ICD versus the ICD group, one significant group effect was found. On physical functioning the ToF + ICD group obtained a higher, more favourable mean score than the ICD group (*p* = 0.01). No other significant differences were found between the three groups.

**Table 2:** Mean scores for the different instruments

	ToF + ICD (*N* = 26)		ToF (*N* = 28)		ICD (*N* = 35)		Normdata			Group effect^a^		
	Median	IQR	Median	IQR	Median	IQR	Mean	SD	*N*	ToF + ICD vs. ToF (*p* value)	ToF + ICD vs. ICD (*p* value)	ToF + ICD vs. Norm (*p* value)
SF-36												
Physical functioning	82.5	[58.8–91.3]	95.0	[76.3–100]	60.0	[40.0–75.0]	93.1	11.7	1,742	**0.01**	**0.01**	**<0.01**
Social functioning	87.5	[62.5–100]	100.0	[75.0–100]	87.5	[62.5–100]	91.2	15.9	1,742	0.1	0.7	**<0.001**
Role limitations due to physical functioning	75.0	[25.0–100]	100.0	[50.0–100]	50.0	[0.0–100]	89.5	24.1	1,742	0.1	0.2	**<0.01**
Role limitations due to emotional functioning	100.0	[66.7–100]	100.0	[75.0–100]	100.0	[66.7–100]	88.9	26.1	1,742	0.5	0.7	0.2
General mental health	72.0	[55.0–85.0]	80.0	[68.0–91.0]	84.0	[72.0–92.0]	81.5	14.1	1,742	0.2	0.1	**<0.01**
Vitality	60.0	[45.0–75.0]	75.0	[50.0–83.8]	65.0	[50.0–75.0]	75.1	15.4	1,742	0.1	0.8	**<0.001**
Bodily pain	82.0	[61.5–100]	100.0	[74.0–100]	84.0	[72.0–100]	84.3	17.3	1,742	0.3	0.8	0.2
General health perceptions	52.0	[38.8–62.0]	69.5	[52.0–82.0]	55.0	[42.0–67.0]	80	14.5	1,742	**<0.01**	0.5	**<0.001**
SWLS	24.0	[13.5–29.0]	28.0	[21.3–31.0]	28.0	[22.0–30.0]	25.5	5	109	**0.02**	**0.03**	**0.03**
LAS	70.0	[68.0–77.8]	80.0	[70.0–86.5]	70.0	[60.0–75.0]	75.4	9	110	0.1	0.4	0.1
HADS												
Anxiety	6.0	[2.0–9.0]	4.0	[3.0–6.0]	2.0	[1.0–5.0]	5.1	3.6	199	0.2	**0.01**	0.4
Depression	2.0	[1.0–3.0]	1.5	[0.0–4.0]	3.0	[2.0–6.0]	3.4	3.3	199	0.8	0.1	0.4
UCL												
Active problem solving	18.0	[14.8–21.0]	19.0	[17.0–21.8]	17.0	[15.0–20.0]	18.6	4	2,205	0.2	0.9	0.2
Palliative reactions	17.5	[15.0–20.0]	16.5	[14.0–20.0]	15.0	[12.0–18.0]	16.1	4.4	2,205	0.3	**<0.01**	**0.03**
Avoiding/waiting	15.5	[13.0–17.0]	16.5	[14.3–18.0]	16.0	[13.0–19.0]	14.9	4.2	2,205	0.2	0.7	0.4
Seeking social support	14.5	[11.8–17.0]	14.5	[13.0–17.0]	11.0	[8.0–13.0]	12.3	3.6	2,205	0.8	**<0.0001**	**<0.01**
Passive reaction pattern	10.0	[9.0–15.3]	10.5	[8.3–12.8]	9.0	[9.0–11.0]	10.8	3.7	2,205	0.4	0.2	0.2
Expression of emotions	6.5	[5.0–8.0]	6.0	[6.0–8.0]	5.0	[4.0–6.0]	6.3	1.9	2,205	0.8	**0.01**	0.3
Reassuring thoughts	12.0	[10.0–14.0]	11.5	[10.0–13.0]	11.0	[9.0–13.0]	11.8	2.9	2,205	0.7	0.2	0.7
IDAS												
Anxiety/fear	21.0	[18.0–29.5]	–	–	19.0	[14.0–24.0]	–	–	–	–	0.1	–
Attitude	9.0	[7.0–12.5]	–	–	9.0	[6.0–10.0]	–	–	–	–	0.2	–
Preparation	8.0	[6.8–10.3]	–	–	7.0	[6.0–11.0]	–	–	–	–	0.5	–
Bodily awareness	6.0	[4.0–8.0]	–	–	5.0	[4.0–7.0]	–	–	–	–	0.2	–
PSSS12												
Significant others	28.0	[23.8–28.0]	28.0	[22.0–28.0]	26.0	[19.0–28.0]	–	–	–	0.6	0.1	–
Family support	24.5	[18.8–27.3]	23.5	[17.3–25.0]	22.0	[16.0–27.0]	–	–	–	0.4	0.2	–
Friends support	21.5	[17.8–25.3]	24.0	[20.0–25.8]	19.0	[16.0–24.0]	–	–	–	0.3	0.4	–
Total score	73.5	[62.5–79.0]	69.5	[65.3–76.0]	65.0	[50.0–78.0]	–	–	–	0.7	0.2	–

ToF + ICD, patients with Tetralogy of Fallot with an ICD

ToF, patients with Tetralogy of Fallot (without an ICD)

ICD, patients with ICD (without congenital heart disease)

*IQR* interquantile range, *SF*-*36* short-form 36, *SWLS* Satisfaction with Life Scale, *LAS* Linear Analogue Scale, *HADS* Hospital Anxiety and Depression Scale, *UCL* Utrecht Coping List, *IDAS* Implanted Devices Adjustment Scale

^a^Group effect: indicates difference between mean ranks between groups, as calculated by a Mann–Whitney *U* test

The bold numbers in the text indicate significant results

#### Satisfaction with Life Scale

Patients of the ToF + ICD group scored significantly lower compared to patients from the ToF group (*p* = 0.02). Also, a significantly lower score was found in the ToF + ICD group compared to the ICD group (*p* = 0.03).

#### Linear Analogue Scale Quality of Life

The ToF + ICD group showed a trend towards a less favourable result than the ToF group (*p* = 0.06).

#### Hospital Anxiety and Depression Scale

Patients from the ToF + ICD group reported significantly more anxiety than patients from the ICD group (*p* = 0.01). No other significant differences were found on the anxiety and depression scale between the three groups.

#### Utrecht Coping List

Patients from the ToF + ICD group scored significantly higher on Palliative reactions (i.e. seeking diversion in unhealthy manners) compared to patients from the ICD group (*p* < 0.01). Also significantly higher scores were found on seeking social support in the ToF + ICD group compared to the ICD group (*p* < 0.0001). Patients from the ToF + ICD group also scored higher on expressions of (negative) emotions compared to the ICD-patients group (*p* = 0.01). No differences in scores were found between the ToF + ICD and the ToF group. No other significant differences were found on the other scales of the UCL.

#### Implanted Devices Adjustment Scale

No significant differences were found between the two ICD groups on any of the IDAS domains.

#### Perceived Social Support Scale

No significant differences were found between the three groups on any of the PSSS12 domains.

### Primary versus secondary indication for ICD implantation

In order to assess whether the indication for ICD implantation had effect on the outcomes, we compared the scores on different scales of patients with a primary indication versus a secondary indication for ICD implantation.

In the ToF + ICD group, no significant differences were found between the two indication groups.

In the ICD group, no significant differences were found between the two indication groups, except for the body awareness scale of the IDAS. Here, patients with a secondary indication showed less favourable outcome.

When combining the ToF + ICD group with the ICD group, a less favourable outcome on the UCL scale in palliative reactions was observed for patients with secondary indication for ICD.

### Normative data

When comparing the ToF + ICD group with normative data, the following results were obtained.

#### Subjective health status (SF36)

Normative data for the Dutch population were obtained from Aaronson et al. [12]. Patients from the ToF + ICD group obtained significantly lower results on physical functioning, social functioning, role limitations due to physical functioning, general mental health, vitality and general health perceptions. No significant difference on the SF-36 scales bodily pain and role limitations due to emotional functioning were found.

#### Satisfaction with Life Scale

Normative data were obtained from Moons et al. [13]. Patients from the ToF + ICD group obtained significantly less favourable outcomes compared to the general Belgium population.

#### Linear Analogue Scale Quality of Life

Normative data were obtained from Moons et al. [13]. No significant differences were found between the ToF + ICD group compared to the normative data.

#### Hospital Anxiety and Depression Scale

Normative data for the HADS have been obtained from Spinhoven et al. [14]. When comparing the ToF + ICD group with normative data, no significant differences were found.

#### Utrecht Coping List

Normative data for the Dutch population were obtained from Schreurs et al. [16]. Patients from the ToF + ICD group obtained significantly less favourable outcomes on palliative reactions and seeking social support.

## Discussion

The main finding of this study is that ToF-patients with an ICD show less favourable psychosocial functioning compared to ToF-patients without ICD and to the older acquired heart disease ICD-patients.

To our knowledge, this is the first psychosocial study carried out in this specific group, Fallot-patients with ICD. Data were compared with two control groups: Fallot-patients without ICD and older “regular” ICD-patients without ConHD. Clinically relevant areas of psychosocial functioning together with medical correlates for psychosocial outcomes were investigated, using standardized and validated questionnaires.

### Psychosocial functioning and ICD therapy

Despite a younger age (40 vs. 72 years) and lower NYHA class (I vs. II), Fallot-patients with ICD scored less favourable on instruments assessing subjective health status, anxiety, satisfaction with life and coping (more negative emotions, more palliative reactions such as smoking and drinking and less seeking of social support) compared to older ICD-patients. After correction for the higher age in the ICD group using stepwise multiple regression, all conclusions drawn remained the same.

In contrast to the overall good quality of life reported before [13], in our study we found that Fallot-patients with ICD showed a less favourable quality of life outcome than Fallot-patients without ICD. Our findings, together with the findings from literature indicate that the ConHD background of these ICD-patients cannot be the sole reason for the lower quality of life observed in this study. In fact, they confirm our hypothesis that ICD therapy in young ConHD patients is associated with worse psychosocial functioning. Between the study of Yap et al. [4] and the start of our own study, patients with less favourable medical outcome have died in the ToF + ICD group. This means that it might even be possible that our present outcomes could have been worse as we face a positive selection of patients.

### Anxiety in Fallot-patients receiving ICD therapy

We found anxiety to be a problematic psychosocial reaction for young Fallot-patients receiving ICD therapy. This is in line with the review of Sears et al. 2009 [19]. Our results on anxiety were statistically significant and in addition clear trends were observed in the other data, also pointing towards the same direction of a less favourable psychological outcome for Fallot-patients with ICD compared to both control groups. In addition, patients reported a lower satisfaction with life.

In literature [20], a clinical cut-off value of 8 is considered clinically significant on the anxiety scale of the HADS. Our ToF + ICD group obtained a median score of 6 and did not show a significant difference on anxiety level compared to normative data. This finding might be explained by assuming that the HADS instrument may not be sensitive enough to screen for disease-specific anxiety in this unique ConHD population. We assume that using a clinical interview, high levels of anxiety might have been found, as in the article of Bromberg et al. [21].

### Role of appropriate and inappropriate ICD shocks

The present data show that Fallot-patients receiving ICD therapy have a higher rate of inappropriate ICD discharges compared to “regular” ICD-patients. Despite optimal programing, 39% of the Fallot-patients with ICD suffered from one or more inappropriate ICD shocks. The inappropriate shock rate was higher than reported in previous studies in non-ConHD patients [3, 5]. Since Fallot-patients are known to have a high arrhythmia burden, the inappropriate shocks we found may be due to atrial arrhythmias [4, 22–24].

Furthermore, ToF ICD-patients are in general younger than the traditional ICD-patient group and tend to lead more active lives, practising sports and other leisure activities. These activities inducing sinus tachycardia may result in inappropriate ICD therapy. Not only the inappropriate shock rate in ToF + ICD patients was higher than that in the older ICD group but also the number of inappropriate shocks per patient was significantly higher. As most inappropriate shocks occur when the patient is fully conscious, this may have serious psychosocial consequences and it may lead to serious anxiety and stress, possibly resulting in avoidance behaviour. Our findings are in line with Vasquez et al. [7], who showed that ICD patients who had a history of more inappropriate shocks with age below 50 and female gender were at higher risk for developing psychosocial problems. Moreover, avoidance behaviour has been reported for ICD patients, which may be a limiting factor in social and sexual activities, but also in practising sports. Out of fear for an ICD discharge, 39% of the ICD-patients avoid physical exertion [25], even though physical exercise is well known to have a beneficial effect on health and can be effective in preventing depression.

Although there is lack of evidence in mortality benefit, the threshold for using ICD therapy in ConHD patients seems to have lowered over time. The guidelines for ICD implantation in this patient population are based on limited data. With the high rate of inappropriate shocks, balancing the benefit-risk ratio for ICD implantation remains difficult, especially taking psychosocial problems into account.

### Medical background

The differences in QRS duration as seen in Table 1 could be explained by the ConHD background in combination with pacemaker therapy differences between groups. ToF-patients have a higher QRS duration as a result of right ventricular dilatation, diminished function, or post-surgery for their ConHD background. Some of the patients in the ToF + ICD group also received constant pacing therapy next to the ICD therapy which may also have resulted into a longer QRS duration. The long QRS duration seen in the ToF + ICD group can also be the result of selection, as a QRS duration >180 ms is a predictor for SCD and may have been used as a criterium for ICD implantation [26]. Despite the diminished RV function, patients in the ToF + ICD group were in good clinical condition with the majority being in NYHA class I.

### Clinical implications

When considering ICD therapy in young patients, the psychosocial impact should be taken into account. The findings in this study provide a solid argument for careful assessment and counselling in patients with Tetralogy of Fallot. The threshold for ICD implantation should be high, especially in case of primary prevention. In patients needing an ICD, routinely applied comprehensive and multidisciplinary psychosocial aftercare is advised. We see an opportunity for a shared decision-making model in this situation. In this way, patients can become well informed about all possible consequences of ICD therapy, and can decide the best treatment option together with the clinician.

In order to facilitate acceptance of ICD therapy, we recommend cognitive behavioural techniques such as psycho-education, cognitive re-appraisal and relaxation techniques to improve quality of life of these patients. These techniques have been found to improve the quality of life in the “general” older ICD population [7, 27, 28].

### Limitations

The patients included in this study were all followed in a tertiary (academic) medical centre. Therefore, this study may not be representative for all Fallot-patients. In addition, although we tried to create comparable groups, some differences were present. Furthermore, because of the small sample size, often encountered in these patient groups, several nearly significant trends were observed. With a larger sample size these trends might have become significant.

Although no significant differences were found between the three groups, a trend was visible in which the older ICD patients more often had male gender.

Unfortunately, data regarding psychosocial interventions (so called “medical consumption” or “psychotherapeutic counselling”) are not systematically available.

Furthermore, as could be expected, a significant age difference was found between the ToF + ICD and the—by definition—older ICD group, which resulted in a later age at implantation in the ICD group. In addition, patients with the Belgian nationality (*N* = 8) were only found in the ToF + ICD group. To which extent these inter-group differences have influenced our results is unknown.

Despite the fact that patients in the ICD group were more often in NYHA class II compared to the ToF and the ToF + ICD group, we remarkably found that our younger NYHA class I ToF + ICD patients obtained less favourable results than the other ICD group with worse NYHA class. This noteworthy finding reflects the psychosocial importance of our results in the ToF + ICD group.

### Future research

Future research should investigate the role of inappropriate shocks on psychosocial outcome in a larger cohort, as the current cohort was not large enough to perform further subanalyses. In addition, the impact of ICD therapy in young adults with ConHD on activities such as practising sports, sexuality and driving a car [29] should be studied. We recommend using a semi-structured clinical interview to assess these points, as questionnaires may not be specific enough.

Different programing strategies, such as the application of antitachycardia pacing therapy and higher rate cut-offs for arrhythmia detection, may prevent inappropriate ICD therapy and may have a beneficial effect on psychosocial functioning.

Recently, the subcutane ICD (sICD) has been introduced for patients requiring ICD therapy [30, 31]. In this study, sICD therapy appeared to have a very low rate of inappropriate shocks. This therefore may be a good alternative for ConHD patients who require ICD therapy and suffer from a lot of inappropriate shocks. The sICD is relatively easy to implant, and because the leads are subcutaneous, replacement and complication rates appear to be lower as well. Future research could concentrate on the application and psychosocial impact of having an sICD in ConHD patients.

## Conclusion

Implantable cardioverter defibrillators implantation has a major psychosocial impact in young adults with ToF. This group shows clinically significant psychosocial problems that have to be recognized and treated appropriately. The information obtained from this study can be used to guide adequate counselling and development of interventions aimed at enhancing psychosocial functioning and improving quality of life. Our results provided information that is not readily apparent from routine clinical investigations.
